# Deep Brain Stimulation of the Nucleus Accumbens in Severe Enduring Anorexia Nervosa: A Pilot Study

**DOI:** 10.3389/fnbeh.2022.842184

**Published:** 2022-04-27

**Authors:** Jessica C. Scaife, John Eraifej, Alexander L. Green, Beth Petric, Tipu Z. Aziz, Rebecca J. Park

**Affiliations:** ^1^Department of Psychiatry, Warneford Hospital, University of Oxford, Oxford, United Kingdom; ^2^Oxford Health NHS Foundation Trust, Oxford, United Kingdom; ^3^Nuffield Department of Surgical Sciences, John Radcliffe Hospital Oxford, University of Oxford, Oxford, United Kingdom; ^4^Oxford University Hospitals NHS Foundation Trust, Oxford, United Kingdom; ^5^Nuffield Department of Clinical Neuroscience, John Radcliffe Hospital, University of Oxford, Oxford, United Kingdom

**Keywords:** clinical trial, anorexia nervosa, deep brain stimulation, compulsivity, reward, treatment

## Abstract

**Introduction:**

Anorexia nervosa (AN) is one of the most debilitating psychiatric disorders, becoming severe and enduring in a third of cases; with few effective treatments. Deep brain stimulation is a reversible, adjustable neurosurgical procedure that has been gaining ground in psychiatry as a treatment for depression and obsessive–compulsive disorder, yet few studies have investigated AN. Abnormal eating behavior and the compulsive pursuit of thinness in AN is, in part, a consequence of dysfunction in reward circuitry and the nucleus accumbens (NAcc) is central to reward processing.

**Methods:**

Phase 1 prospective open-label pilot study of seven individuals with severe enduring AN. Electrodes were implanted bilaterally into the NAcc with stimulation at the anterior limb of the internal capsule using rechargeable implantable pulse generators. The protocol of 15 months included 12 months of deep brain stimulation incorporating two consecutive, randomized blind on-off fortnights 9 months after stimulation onset. The primary objectives were to investigate safety and feasibility, together with changes in eating disorder psychopathology.

**Results:**

Feasibility and safety was demonstrated with no serious adverse events due to deep brain stimulation. Three patients responded to treatment [defined as > 35% reduction in Eating Disorders Examination (EDE) score at 12 months] and four patients were non-responders. Responders had a statistically significant mean reduction in EDE scores (50.3% reduction; 95% CI 2.6–98.2%), Clinical Impairment Assessment (45.6% reduction; 95% CI 7.4–83.7%). Responders also had a statistically significant mean reduction in Hamilton Depression Scale, Hamilton Anxiety Scale and Snaith-Hamilton pleasure scale. There were no statistically significant changes in Body Mass Index, Yale-Brown-Cornell Eating Disorder Scale, Yale-Brown Obsessive–Compulsive Scale and World Health Organization Quality of Life Psychological subscale.

**Conclusion:**

This study provides some preliminary indication that deep brain stimulation to the NAcc. Might potentially improve some key features of enduring AN. In this small study, the three responders had comorbid obsessive-compulsive disorder which predated AN diagnosis. Future studies should aim to further elucidate predictors of outcome.

**Clinical Trial Registration:**

[www.ClinicalTrials.gov], identifier [Project ID 128658].

## Introduction

Anorexia nervosa (AN) is a severe psychiatric disorder with an incidence of 0.9–4% in females and 0.3% in males in the population (Zipfel et al., 2015; Martínez-González et al., 2020). The prognosis is poor: around one third of individuals develop severe and enduring AN (Arcelus et al., 2011). There is a huge need for novel treatments for patients who have found no beneficial psychological or pharmacological treatment (Crow et al., 2009; Balestrieri et al., 2013; Watson and Bulik, 2013; Hay et al., 2021). Neurosurgical interventions may offer a solution but must meet ethically high standards and should be reversible unlike some proposed lesion treatments (Park et al., 2017; Pugh et al., 2018; Pugh, 2019).

Deep brain stimulation is a reversible, adjustable and invasive neurosurgical procedure, in which electrodes are inserted into specific neural targets. Originally developed by Heath for use in psychiatric disorders (O’Neal et al., 2017) and later pain (Hosobuchi et al., 1977; Richardson and Akil, 1977), its main use currently is for movement disorders (Krack et al., 2019). More recently, its use in psychiatric disorders has enjoyed a renaissance (Cleary et al., 2015; Graat et al., 2017): obsessive–compulsive disorder (OCD) (de Haan et al., 2017; Bergfeld et al., 2021) and it has been used in depression (Malone et al., 2009; Bewernick et al., 2010) and addiction (Wang et al., 2018; Vannemreddy and Slavin, 2019). Most studies targeted the ventral striatum (VS)/nucleus accumbens (NAcc) (Malone et al., 2009; Bewernick et al., 2010) and subcallosal cingulate (Lozano et al., 2008, 2012; Lipsman et al., 2017).

The NAcc in the VS is a locus of hedonic pleasure and reward learning (Hill et al., 2014; Berridge and Kringelbach, 2015). Compulsivity is a trans-diagnostic feature of both OCD and AN (Godier and Park, 2014, 2015). In both, dysfunctional cortical-striatal thalamic reward pathways (Lipsman et al., 2015) allow habitual behavior mediated by structures in the dorsal striatum to take over (Robbins and Everitt, 1996; Steinglass and Walsh, 2006; Haber and Knutson, 2010; Simmler and Ozawa, 2019).

In prior studies of patients with OCD, deep brain stimulation has shown some efficacy when targeted at the anterior limb of the internal capsule, NAcc/VS/ventral capsule and the subthalamic nucleus (Greenberg et al., 2010; Kohl et al., 2015; Senova et al., 2019). Given the paucity of effective treatments for severe AN there is growing interest in the use of brain stimulation techniques such as transcranial magnetic stimulation, transcranial direct current stimulation and deep brain stimulation as possible treatments (Lozano et al., 2019; Duriez et al., 2020). There have been only a handful of prior studies of deep brain stimulation in AN. Lipsman et al. (2017) targeted the subcallosal cingulate with a 50% response rate at 1 year (Lipsman et al., 2017) and no further improvements at 3 years follow-up with a focus on body mass index (BMI) as a measure of “recovery.” Two recent deep-brain stimulation (DBS) case series targeted the NAcc (Liu et al., 2020; Villalba Martínez et al., 2020) and both focused on BMI as a primary outcome measure. Neither of these two studies reports on eating disorder psychopathology and no prior study in AN has included a blind on vs. off period, making it impossible to establish changes in eating disorder psychopathology, or if any effects are a genuine attributable to DBS.

Our study attempts to overcome these important limitations by inclusion of a rigorously assessed Eating disorder psychopathology as the main primary outcome measure and a blind DBS on off period, alongside a rigorous ethical gold standard (Park et al., 2017). We included only patients with severe, enduring anorexia nervosa (SE-AN) who had exhausted other treatment options over decades. We chose to target the NAcc because of its efficacy in other compulsive disorders. We used rigorously assessed eating disorder psychopathology as the main primary outcome measure rather than solely BMI, as body weight is subject to change due to inpatient regimes, and/or the onset of binges. It does not necessarily reflect change in underlying eating disorder psychopathology.

## Materials and Methods

### Aims and Outcomes

This study aimed firstly to demonstrate that it was feasible and safe to perform DBS surgery in malnourished patients with severe AN. Adverse events associated with surgery or stimulation were recorded post-operatively in electronic patient records at monthly follow up. Self-reported side effects were recorded in side-effect questionnaires, the SAFTEE-SI (Clyde, 1986) and the DBS side effects questionnaire (Brunoni et al., 2011).

Secondary aims of the study were to assess the effect of DBS to the NAcc on core eating disorder psychopathology, assessed using the current gold standard semi structured interview Eating Disorder Examination (EDE) (Fairburn et al., 2008), together with The Clinical Impairment Assessment CIA (Bohn et al., 2008), the Yale-Brown-Cornell Eating Disorder Scale: (YBC-EDS) (Mazure et al., 1994) and BMI. Food preference was also assessed behaviorally, using the Leeds food preference questionnaire (Finlayson et al., 2007; Cowdrey et al., 2013). Separate measures of liking (hedonic pleasure) and explicit wanting (incentive salience) were assessed by using food stimuli varying along the dimensions of calorie content (high or low) and taste (savory or sweet). Explicit wanting and liking were assessed using 100 mm VAS scales, responding to the questions “How much do you want some of this food now?” and “How pleasant would it be to experience the taste of this food now?”, respectively. Implicit wanting was indexed using reaction times to a behavioral forced-choice component of the task.

Tertiary outcomes were to assess comorbid psychopathologies: OCD using the Yale-Brown Obsessive–Compulsive Scale (YBOCS) (Goodman et al., 1989); anxiety using the observer-rated Hamilton Anxiety Rating Scale (HAMA) (Hamilton, 1959), and depression using the observer rated Hamilton Depression Response Scale (HAM-D; Hamilton, 1960). Anhedonia and life-quality were assessed using the Snaith-Hamilton pleasure scale (SHAPS) (Snaith et al., 1995) and the World Health Organization Quality of Life Scale (WHOQUAL-BREF), psychological subscale (Skevington, 1999). Global Assessment of Functioning (GAF) Diagnostic and Statistical Manual of Mental Disorders, fourth edition (American Psychiatric Association [APA], 1980). For full details of study assessments, see our published protocol paper (Park et al., 2018).

### Inclusion/Exclusion Criteria

Key inclusion criteria were: age: 21–60 years, clinical diagnosis of restrictive AN (DSM-5 criteria), duration enduring > 7 years, and treatment-resistance (defined as lack of response to ≥3 voluntary intensive treatments (partial or full hospitalization) and at least 2 trials of psychological treatment) preoperative BMI between 13 and 16 (one participant exceeded this as she was an inpatient on a meal-plan); and capacity to fully understand the study and to provide informed consent. Key exclusion criteria were: current or past psychotic episode, comorbid neurological illness, involuntary treatment or drug abuse in the last year, contraindications to undergo magnetic resonance imaging, any medical condition involving a risk for the surgical procedure, and pregnancy.

Written informed consent was obtained from all participants before proceeding with any intervention. This study will be carried out in accordance with the recommendations of NRES: South Central—Oxford A Research Ethics Committee (REC) Ref: 13/SC/0267. The study was performed according to the ethical standards stated in the Declaration of Helsinki and subsequent updates. Our published protocol paper gives full details on the patient selection process (Park et al., 2018).

### Surgery

**FIGURE 1:**
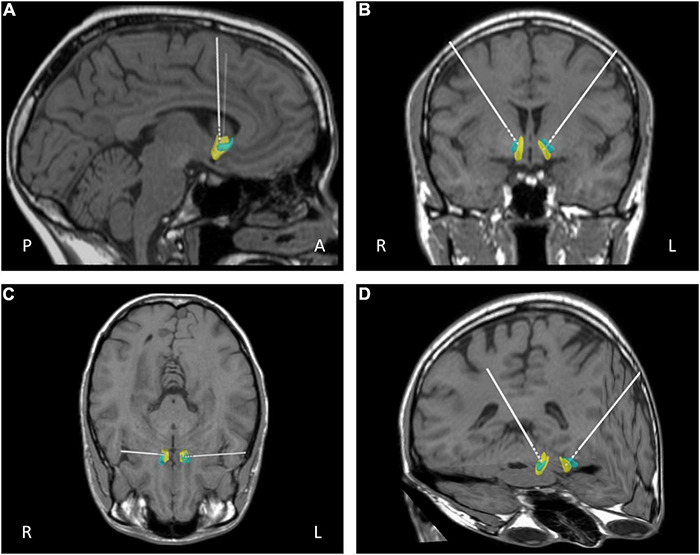
Representative post-operative reconstruction of DBS electrode in native patient space with nucleus accumbens (NAcc) mask (Cartmell et al., 2019): A, anterior; P, posterior; R, right; L, left; blue, core; yellow, shell. **(A)** Sagittal view. **(B)** Coronal view. **(C)** Axial view **(D)** 3D reconstruction.

Surgery was performed under general anesthesia. A 2.7 mm twist drill craniotomy was made and the electrode lead inserted bilaterally into the NAcc shell. All patients have intra-operative imaging to confirm electrode positioning is within the target and repositioned in real time if not. All electrodes were confirmed using fused pre-op MRI fused with postoperative CT with distal contact in NAcc and proximal contacts in ALIC. Target selection was based on anatomical/stereotactic references, for a representative picture of implantation, see Figure 1. For further details, see published paper (Park et al., 2018).

Two different models of stimulator were used: For patients 1, 2, 5, 6 and 7 a constant voltage stimulator was used: Medtronic Activa RC model 37612. For patients 3 and 4 a constant current stimulator was used: Boston Scientific Vercise RC IPGs model DB-1110-C. Despite the differences in stimulator model, the electrodes were identical in design (similar diameter, circumferential contacts with similar electrical parameters). Furthermore, there is no evidence that differences in implanted device affects outcome in other diseases such as Parkinson’s or tremor. The more important variable here is that the stimulation parameters are uniform across participants which is the case here (with up titration of voltage over time).

After implantation, the DBS was programmed by a trained clinician to optimize symptom suppression and control side effects. The day after surgery, the stimulator was turned on to ascertain any immediate effects of stimulation on symptoms and then switched off again. The stimulator was switched on following all wound healing, one month following the operation. Bipolar stimulation was delivered at 130 Hz and voltage/current was adjusted over subsequent visits to a maximum which was titrated to the maximum tolerated each patient. See Table 1 for the final stimulator settings.

**TABLE 1:** Demographic and clinical patient characteristics.

Patient	Sex	Age	Illness duration (years)	BMI (historic low)	Psychiatric comorbidities	Psychotropic medication at time of surgery	Prior inpatient admissions	Medical complications	Maximum voltage (V)
1	F	54	40	13.0	OCD MDD	Venlafaxine	>3	Osteoporosis	4.0
2	F	26	13	12.0	OCD	None	>4	Osteoporosis	4.0
3	F	28	14	13.0	OCD MDD GAD	Sertraline Mirtazapine Pregabalin	>4	Osteoporosis	3.75
4	M	38	12	12.0	OCPD Severe recurrent MDD	None	>5	Leukopenia abnormal LFT	4.0
5	F	58	36	14.0	MDD	Venlafaxine	>3	Osteoporosis	4.0
6	F	25	15	13.0	OCD	None	>4	Osteoporosis	3.5
7	F	30	17	11.0	MDD	Sertraline	>5	Osteoporosis Leukopenia	4.5
Mean (*SD*)		37 (13.7)	21 (11.8)	12.6 (1.0)					

*BMI, Body mass index; OCD, Obsessive–Compulsive Disorder; MDD, Major Depressive Disorder; GAD, Generalized Anxiety Disorder; OCPD, Obsessive Compulsive Personality Disorder; LFT, Liver function tests. Mean age: 38 ± 12.9 years. Mean illness duration: 12 ± 21 years.*

*Mean BMI: 12.6 ± 1. Medication at end of protocol was the same as at time of surgery, but also Patient 5 on Fluoxetine and Patient 2 on Fluvoxamine. Stimulation parameters: all patients were stimulated at 130 Hz.*

### Follow-Up

During the protocol period patients were followed up monthly for 12 months after DBS was switched on. They were has joint psychiatric/neurosurgical reviews monthly and had a separate assessment of psychopathology using semi structured interview (EDE and self-report schedules; Park et al., 2018). Approximately 1 month after surgery, patients were reviewed by a senior consultant psychiatrist and consultant neurosurgeon prior to DBS switch-on. The battery of tests taken at this time point was used as baseline for data analysis.

After approximately 9 months after DBS switch on, participants had a blind on/off month where they received 2 weeks on or 2 weeks off (randomized in order) and blinded to the condition. Three un-blinded clinicians (TA, RP, BP) adjusted the stimulation parameters. Participants and other team members, in particular the assessor of EDE and all psychopathology measures (JCS) were blinded to on or off condition. All patients received treatment as usual from a separate NHS clinical team, alongside the DBS protocol. Note that Patient 5 underwent the nested double-blind study at six months, Patient 6 did so at 10 months and Patient 7 did so outside of the protocol period (at 15 months from baseline) due to the COVID-19 pandemic.

### Statistical Analysis

Mean percentage reduction in eating disorder pathology and psychometric test scores were calculated and a 95% confidence interval for this presented. This was done at a group level and for responders and non-responders respectively. Responder status was defined as >35% reduction in EDE in accordance with the previously published literature in OCD and AN (Denys et al., 2010, 2020; Lipsman et al., 2017). The effect of time on outcome was calculated using a two-way repeated measures ANOVA and results of the nested double-blind study were analyzed using a paired *t*-test. Statistical significance level was set at *p* < 0.05 and all analysis was completed in SPSS 28 (IBM).

## Results

### Participants Characteristics

Seven patients (six female) were enrolled into this pilot study (Table 1). Mean age at surgery was 37 (13.7) and mean duration of illness was 21 years (SD 11.8). Mean historic BMI was 12.6 (SD 1.0). Patient 3 had an immediate pre-operative BMI of 18 as she was an inpatient by the time of operation, which was delayed as she sustained a fracture in the preoperative period. By the time of operation, she had undergone intensive weight restoration from a lower BMI at the time of recruitment, but was unable to eat outside of an inpatient setting. All patients experienced at least 3 inpatient admissions, and failed numerous treatments prior to inclusion in the study. All participants had co-morbid psychiatric disorders. This included five with comorbid major depressive disorder (MDD) and four with obsessive–compulsive disorder (OCD) (Table 1). All patients received stimulation at 130 Hz with a maximum voltage of 4.5 V.

### Adverse Events

Deep-brain stimulation surgery was well tolerated in all patients. No serious adverse events occurred within the trial period (Table 2) and all patients elected to retain the stimulators at the end of the protocol, continuing to charge them. In two patients side effects limited dose increases: Patient 3 experienced some facial “tingling” when the stimulator was increased to 4.0 V with no motor symptoms, and thus remained on 3.75 V until near the end of the protocol, when she felt she wanted try a higher voltage. Patient 6 experienced nausea when her stimulators where turned up to 4 V, which ameliorated when it was reduced to 3.75 V. Side effects prompted by a side effects questionnaire are detailed in Supplementary Table 1: notably, memory impairment in patient 6 was subjective and not supported by neuropsychological testing pre/post DBS^1^.

**TABLE 2:** Adverse events table at 15 months: Adverse events defined as any complication, expected or unexpected, reported in the electronic patient record in the post-operative period.

Patient	Serious adverse events	Other adverse events
1	None	None
2	None	None
3	None	Sensation of facial tightness with right sided electrode testing (no facial pulling)
4	None	None
5	None	Shallow right frontal extradural hematoma (asymptomatic and treated conservatively, not requiring surgery)
6	None	Wound revision at 3 months under local anesthesia. Nausea at high voltage.
7	None	Possible intra-operative seizure

### Eating Disorder Psychopathology

**FIGURE 2:**
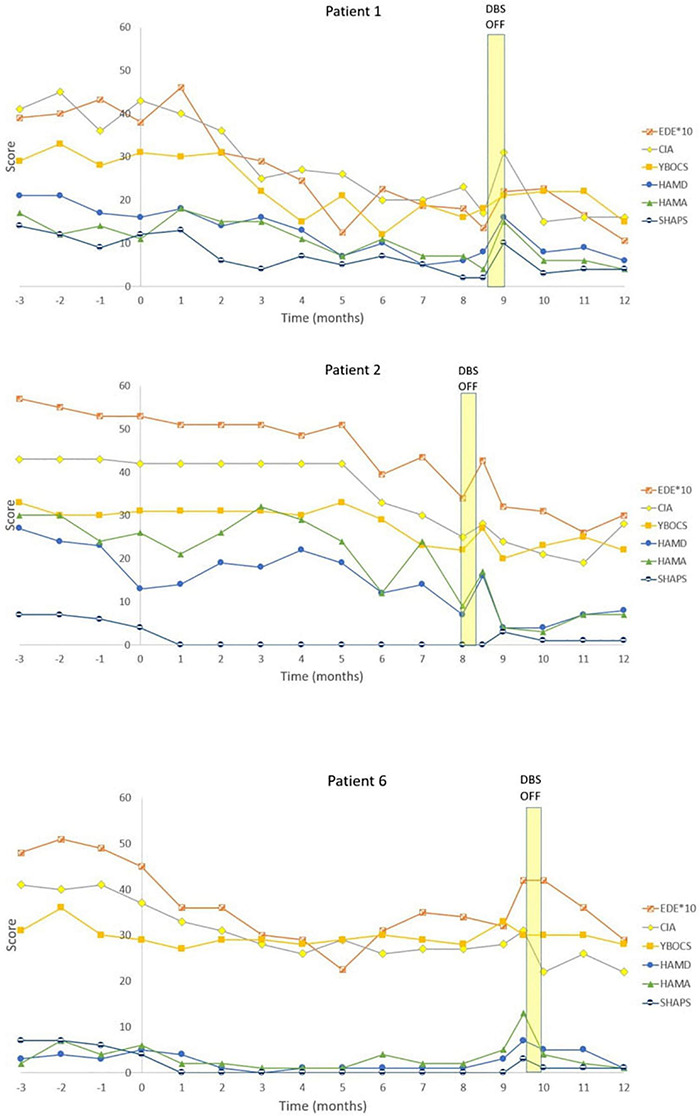
Individual graphs for Responders: Patients 1, 2, 6. Eating Disorder Examination (EDE) X 10 for graphical purposes, Yale-Brown-Cornell Eating Disorder Scale (YBC_EDS), Hamilton Depression Scale (HAMD), Hamilton Anxiety Scale (HAMA), Snaith-Hamilton Pleasure Scale (SHAPS): Yale-Brown Obsessive-Compulsive Scale (YBOCS).

**FIGURE 3:**
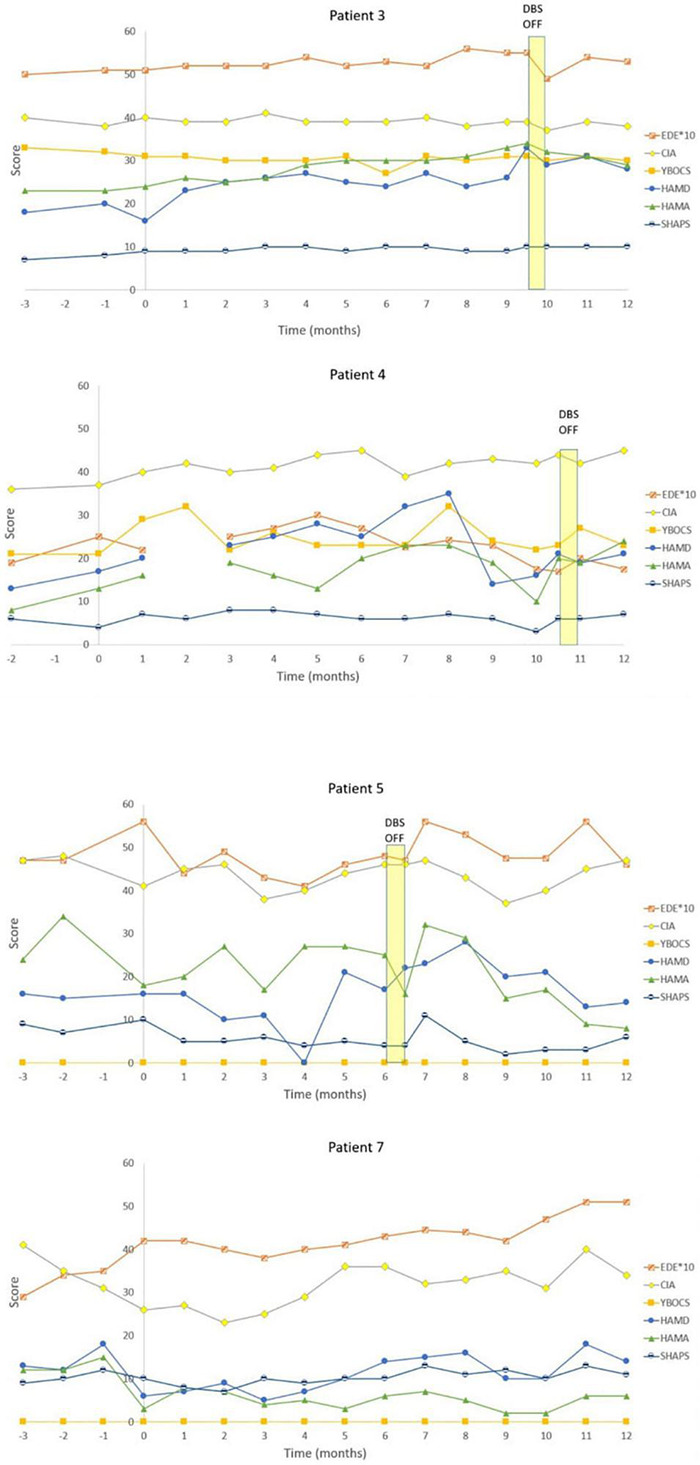
Individual graphs for Non-responders: Patients 3, 4, 5, and 7. Eating Disorder Examination (EDE) X 10 for graphical purposes, Yale-Brown-Cornell Eating Disorder Scale (YBC_EDS), Hamilton Depression Scale (HAMD), Hamilton Anxiety Scale (HAMA), Snaith-Hamilton Pleasure Scale (SHAPS): Yale-Brown Obsessive-Compulsive Scale (YBOCS).

**FIGURE 4:**
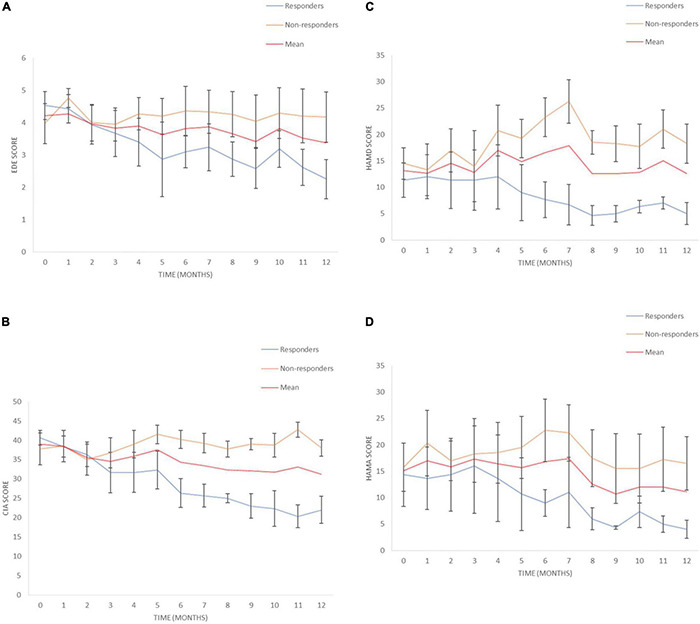
Eating disorders pathology over time. **(A)** Eating Disorder Examination (EDE) scores for responders and non-responders. **(B)** Clinical Impairment Assessment (CIA) scores for responders and non-responders. **(C)** Hamilton Depression Scale (HAMD) scores for responders and non-responders, **(D)** Hamilton Anxiety Scale (HAMA) scores for responders and non-responders. Mean and SEM presented.

Eating disorder examination (EDE) scores reduced by a mean of 19.9% [95% Confidence Interval (CI) from –9.9 to 49.7%] for all patients at 12 months (Table 3). Responder status was defined as >35% reduction in EDE in accordance with the previously published literature in OCD and AN (Denys et al., 2010, 2020; Lipsman et al., 2017). Three patients met responder criteria and four patients did not. Amongst the responders, there was a statistically significant reduction in EDE of 50.3% (95% CI 2.6–98.2%). Amongst non-responders, mean reduction was –2.9% (95% CI –25.9 to 20.1%). Clinical Impairment Assessment (CIA) scores had a mean reduction of 17.3% (95% CI –12.2 to 46.7%) at 12 months (Table 3). Responders had a statistically significant mean reduction of 45.6% (95% CI 7.4–83.7%) whilst non-responders had a mean reduction of –3.9% (95% CI –38.4 to 30.5%). Yale-Brown-Cornell Eating Disorder Scale (YBC-EDS) scores had a mean reduction of 7.9% (95% CI –15.7 to 31.5%) after 12 months (Table 3). Responders had a mean reduction of 31.1% (95% CI –12.4 to 74.6) whilst non-responders had a mean reduction of –9.5% (95% CI 29.3–10.3%). See individual graphs in Figures 2, 3 and group means graphed in Figure 4.

**TABLE 3:** Eating disorder psychopathology scores: EDE, Eating Disorder Examination; CIA, Clinical Impairment Assessment; YBC-EDS, Yale-Brown-Cornell Eating Disorder Scale; BMI, Body Mass Index.

Patient	Baseline	3 Months	6 Months	12 Months	Reduction (%)
**EDE**					
1	**3.8**	**2.9**	**2.25**	**1.06**	**72.1**
2	**5.3**	**5.1**	**3.95**	**3**	**43.4**
3	5.1	5.2	5.3	5.3	–3.9
4	2.2	2.7	2.26	1.9	13.6
5	4.4	4.1	5.6	4.4	0
6	**4.5**	**3**	**3.1**	**2.9**	**35.6**
7	4.2	3.8	4.3	5.1	–21.4
Mean (*SD*)	4.2 (1.0)	3.8 (1.0)	3.8 (1.4)	3.4 (1.6)	19.9 (32.2)
**CIA**					
1	**43**	**25**	**20**	**16**	**62.8**
2	**42**	**42**	**33**	**28**	**33.3**
3	40	41	39	38	5.0
4	40	41	39	44	–10.0
5	45	40	47	36	20.0
6	**37**	**28**	**26**	**22**	**40.5**
7	26	25	36	34	–30.8
Mean (*SD*)	39.0 (6.3)	34.6 (8.1)	34.3 (9.0)	31.1 (9.7)	17.3 (31.8)
**YBC-EDS**					
1	**24**	**18**	**9**	**12**	**50.0**
2	**25**	**25**	**21**	**18**	**28.0**
3	27	30	30	29	–7.4
4	19	24	28	23	–21.1
5	18	11	12	21	–16.7
6	**26**	**30**	**25**	**22**	**15.4**
7	14	11	18	13	7.1
Mean (*SD*)	21.9 (4.9)	21.3 (8.1)	20.4 (7.9)	19.7 (5.9)	7.9 (25.5)
**BMI**					
1	**14.9**	**15.5**	**15.9**	**15.8**	**–6.0**
2	**15.2**	**16.6**	**16.2**	**16.9**	**–11.2**
3	18	15.1	16.1	17	5.6
4	13.7	14.1	13.4	13.8	–0.7
5	15.9	15.6	15.8	16.4	–3.1
6	**14.8**	**14.2**	**13.8**	**14**	**5.4**
7	13.6	15.3	13.7	13.5	0.7
Mean (*SD*)	15.2 (1.5)	15.2 (0.9)	15.0 (1.3)	15.3 (1.5)	–1.3 (6.1)

*Responders’ scores in bold.*

Body mass index had a mean increase of 1.3% (95% CI –4.3 to 6.9%). Responders had a mean increase in BMI of 3.9% (95% CI –17.2 to 25.0%) compared to non-responders that had a mean reduction of –0.6% (95% CI –6.4 to 5.2%) (Table 3).

### Compulsivity

Yale-Brown Obsessive–Compulsive Scale (YBOCS) score had a mean reduction of 13.5% (95% CI –4.7% to 31.6%) at 12 months. Responders had a mean reduction of 28.0% (95% CI –31.8 to 87.9%) compared to non-responders who had a mean reduction of 2.5% (95% CI –2.7 to 7.8%) (Table 4).

**TABLE 4:** Psychometric scores: HAMD, Hamilton Depression Scale; HAMA, Hamilton Anxiety Scale; WHO-QOL-PSYCH, World Health Organization Quality of Life Psychological Subscale; SHAPS, Snaith-Hamilton Pleasure Scale; YBOCS, Yale-Brown Obsessive-Compulsive Scale.

Patient	Baseline	3 Months	6 Months	12 Months	Reduction (%)
**YBOCS**					
1	**31**	**22**	**12**	**15**	**51.6**
2	**31**	**31**	**29**	**22**	**29.0**
3	31	30	27	30	3.2
4	29	26	23	27	6.9
5	0	0	0	0	0.0
6	**29**	**29**	**30**	**28**	**3.4**
7	0	0	0	0	0.0
Mean (*SD*)	21.6 (14.8)	19.7 (13.8)	17.3 (13.2)	17.42 (12.9)	13.5 (19.6)
**HAMD**					
1	**16**	**16**	**10**	**6**	**62.5**
2	**13**	**18**	**12**	**8**	**38.5**
3	16	26	24	28	–75.0
4	20	25	32	20	0.0
5	16	0	23	11	31.3
6	**5**	**0**	**1**	**1**	**80.0**
7	6	5	14	14	–133.3
Mean (*SD*)	13.1 (5.6)	12.9 (11.2)	16.6 (10.4)	12.6 (9.0)	17.3 (31.8)
**HAMA**					
1	**11**	**15**	**11**	**4**	**63.6**
2	**26**	**32**	**12**	**7**	**73.1**
3	24	26	30	29	–20.8
4	16	16	23	20	–25.0
5	20	27	32	11	45.0
6	**6**	**1**	**4**	**1**	**83.31**
7	3	4	6	6	–100.0
Mean (*SD*)	15.1 (8.8)	17.3 (11.8)	16.9 (11.4)	11.1 (10.0)	17.0 (67.6)
**SHAPS**					
1	**12**	**4**	**7**	**4**	**66.7**
2	**8**	**7**	**1**	**0**	**100.0**
3	9	10	10	10	–11.1
4	7	8	6	7	0.0
5	5	4	11	6	–20.0
6	**4**	**0**	**0**	**1**	**75.0**
7	10	10	10	11	–10.0
Mean (*SD*)	7.9 (2.8)	6.1 (3.7)	6.4 (4.4)	5.6 (4.2)	28.7 (49.9)
**WHO-QOL PSYCH**					
1	**7**	**9**	**9**	**11**	**–57.1**
2	**6**	**6**	**11**	**12**	**–100.0**
3	12	8	8	9	25.0
4	5	5	5	5	0.0
5	7	7	6	7	0.0
6	**9**	**10**	**11**	**13**	**–44.4**
7	9	10	9	9	0.0
Mean (*SD*)	7.9 (2.3)	7.9 (2.0)	8.4 (2.3)	9.4 (2.8)	–25.2 (43.6)

*Responders’ scores in bold.*

### Mood and Quality of Life

There was a mean reduction in Hamilton Depression Scale (HAMD) of 0.6% (95% CI –71.2 to 72.4%) at 12 months (Table 4). Responders had a statistically significant mean reduction in HAMD of 60.3% (95% CI 8.5–112.1%) whilst non-responders had a mean increment of +4 4.3% (95% CI –162.45 to 73.9%). There was a mean reduction in Hamilton Anxiety Scale (HAMA) of 17.0% (95% CI –45.5 to 79.5%) (Table 4). Responders had a statistically significant mean reduction in HAMA of 73.3% (95% CI 48.9–97.8%) compared to non-responders who had a mean reduction of –25.2% (95% CI –119.5 to 69.1%). There was a mean reduction in the Snaith-Hamilton pleasure scale (SHAPS) of 28.7% (95% CI –17.5 to 74.8%) (Table 4). Responders had a statistically significant reduction in SHAPS of 80.6% (95% CI 37.5–123.6%) whilst non-responders had a mean reduction of –10.3% (95% CI –23.3 to 2.7%). See individual graphs in Figures 2, 3 and group means graphed in Figure 4.

Quality of life, measured by the World Health Organization Quality of Life Psychological subscale (WHO-QOL-Psych) had a mean improvement of 25.2% (95% CI –15.1 to 65.6%). Responders had a mean improvement of 67.2% (95% CI –5.1 to 139%) whilst non-responders had a mean improvement of –6.25% (95% CI –26.1 to 13.6%).

### Effect of Time on Outcome

In a repeated measures ANOVA, there was a significant effect of time on reduction in EDE (*p* = 0.012), CIA (*p* = 0.009) and WHO-QOL-PSYCH (*p* = 0.016) (Tables 5, 6). Observed power can be observed for all metrics.

**TABLE 5:** Change in eating disorder pathology over time.

	Baseline		3 Months		6 Months		12 Months		Repeated measures ANOVA			
	Mean	*SD*	Mean	*SD*	Mean	*SD*	Mean	*SD*	Mauchly P	*F*	*p*	Observed power
**EDE**												
Responder	4.53	0.75	3.67	1.24	3.10	0.85	2.25	1.04	0.216	5.105	**0.012**	0.832
Non-responder	3.98	1.24	3.95	1.03	4.37	1.51	4.18	1.56				
**CIA**												
Responder	40.67	3.21	31.67	9.07	26.33	6.51	22.00	6.00	0.786	5.542	**0.009**	0.864
Non-responder	37.75	8.18	36.75	7.85	40.25	4.72	38.00	4.32				
**YBC-EDS**												
Responder	25.00	1.00	24.33	6.03	18.33	8.33	17.33	5.03	0.325	1.014	0.414	0.222
Non-responder	19.50	5.45	19.00	9.56	22.00	8.49	21.50	6.61				
**BMI**												
Responder	14.97	0.21	15.43	1.20	15.30	1.31	15.57	1.46	0.142	0.248	0.861	0.087
Non-responder	15.30	2.09	15.03	0.65	14.75	1.40	15.18	1.78				

*EDE, Eating disorder examination; CIA, Clinical Impairment Assessment; YBC-EDS, Yale-Brown-Cornell Eating Disorder Scale; BMI, Body Mass Index.*

*Repeated measures Analysis of Variance (ANOVA) completed for each eating disorder metric according to responder status.*

*Responders’ scores in bold.*

**TABLE 6:** Change in psychometric scores over time.

	Baseline		3 Months		6 Months		12 Months		Repeated measures ANOVA			
	Mean	*SD*	Mean	*SD*	Mean	*SD*	Mean	*SD*	Mauchly P	*F*	*P*	Observed power
**YBOCS**												
Responder	30.33	1.15	27.33	4.73	23.67	10.12	21.67	6.51	0.006	3.457	0.105*	0.384
Non-responder	15.00	17.34	14.00	16.25	12.50	14.53	14.25	16.50				
**HAMD**												
Responder	11.33	5.69	11.33	9.87	7.67	5.86	5.00	3.61	0.168	0.744	0.542	0.172
Non-responder	14.50	5.97	14.00	13.44	23.25	7.37	18.25	7.50				
**HAMA**												
Responder	14.33	10.41	16.00	15.52	9.00	4.36	4.00	3.00	0.167	2.216	0.129	0.452
Non-responder	15.75	9.11	18.25	10.72	22.75	11.81	16.50	10.15				
**SHAPS**												
Responder	8.00	4.00	3.67	3.51	2.67	3.79	1.67	2.08	0.506	2.396	0.109	0.485
Non-responder	7.75	2.22	8.00	2.83	9.25	2.22	8.50	2.38				
**WHO-QOL-PSYCH**												
Responder	7.33	1.53	8.33	2.08	10.33	1.15	12.00	1.00	0.123	4.741	**0.016**	0.801
**Non-responder**	8.25	2.99	7.50	2.08	7.00	1.83	7.50	1.91				

*HAMD, Hamilton Depression Scale; HAMA, Hamilton Anxiety Scale; WHO-QOL-PSYCH, World Health Organization Quality of Life Psychological subscale; SHAPS, Snaith-Hamilton Pleasure Scale; YBOCS, Yale-Brown Obsessive-Compulsive Scale.*

Repeated measures ANOVA completed for each psychomotor score according to responder status. *Greenhouse–Geisser correction since sphericity assumption not met. Responders’ scores in bold.

### Double-Blind ON/OFF Results

A statistically significant mean reduction in HAMD score of 5 (95% CI 0.4–9.6) was noted with DBS on compared to DBS off (*p* = 0.037) (see Table 7). Other pairwise comparisons were non-significant.

**TABLE 7:** Difference in psychometric scores during double-blind period (DBS OFF – DBS ON).

		95% Confidence		Paired samples		
		Interval		*t*-test		
Psychometric score	Mean difference	Lower	Upper	*t*	df	*P* (two-tailed)
EDE	0.1	–0.5	0.7	0.563	6	0.594
CIA	2.0	–4.4	8.4	0.766	6	0.472
YBC_EDS	0.9	–1.4	3.1	0.915	6	0.395
YBOCS	1.6	–0.9	4.0	1.577	6	0.166
HAMD	5.0	0.4	9.6	2.664	6	**0.037**
HAMA	1.6	–8.5	11.6	0.382	6	0.716
SHAPS	1.0	–3.3	5.3	0.568	6	0.590

*HAMD, Hamilton Depression Scale; HAMA, Hamilton Anxiety Scale; WHO-QOL-PSYCH, World Health Organization Quality of Life Psychological subscale; SHAPS, Snaith-Hamilton Pleasure Scale; YBOCS, Yale-Brown Obsessive–Compulsive Scale.*

Repeated measures ANOVA completed for each psychomotor score according to responder status. *Greenhouse–Geisser correction since sphericity assumption not met. Responders’ scores in bold.

### Leeds Food Preference Task

#### Responders

**FIGURE 5:**
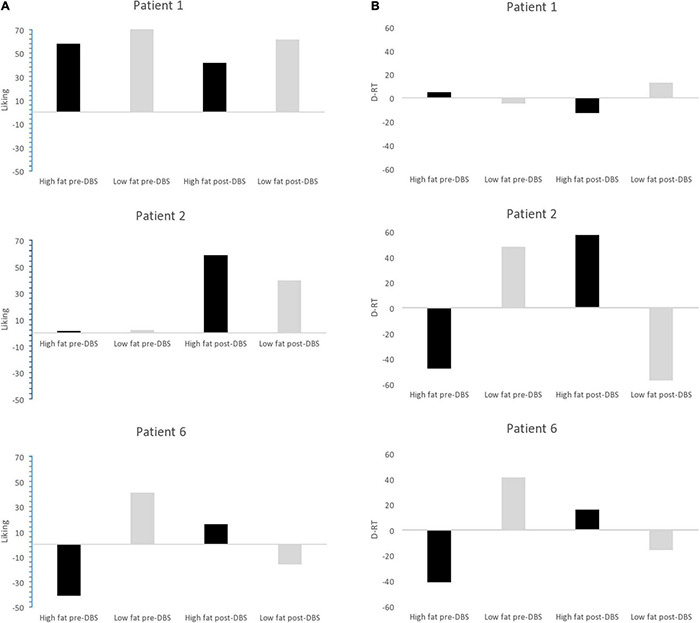
**(A)** Liking scores for high fat and low fat food pre-DBS and post-DBS in responders. **(B)** Implicit wanting DRT scores for high fat and low fat food pre-DBS and post-DBS in responders.

Patient 1’s liking for high-fat food was 58 (out of 100) at baseline and 42 at the end of the protocol. Her implicit wanting score reduced by 16 (56 to 40) indicating slower/less frequent choice of high-fat food by the end of the protocol. See Figure 5. Patient 2 began the study with extremely low liking for high fat foods (1 out of 100) and by the end of the protocol, scores for high fat foods had increased to 61. Her implicit wanting score was strongly in the direction of low-fat over high-fat foods at baseline (–48), and her scores reversed toward high-fat food by the end of the protocol (+57). This represented a change of 105 indicating faster/more frequent choice for high fat food. See Figures 5A,B.

Patient 6 increased by 16 pre-post DBS on liking for high fat foods (1–16) and her implicit wanting score showed an increase of 56 (–41 to 16 pre-post) indicating faster/more frequent choice for high fat food by the end of the protocol. See Figure 5.

#### Non-responders

In all non-responders there was a decrease in liking for high-fat foods ranging from –1 to -24, implicit wanting was also decreased for high-fat foods in. Results in Table 8.

**TABLE 8:** High fat and low fat are collapsed across sweet and savory categories.

Patient number	Preoperative		Pre-switch on		Double-blind On		Double-blind Off		End of protocol	
	Liking High Fat	Liking Low Fat	Liking High Fat	Liking Low Fat	Liking High Fat	Liking Low Fat	Liking High Fat	Liking Low Fat	Liking High Fat	Liking Low Fat
1	52.8	65.4	57.8	74.5	54.6	65.1	32.4	59.4	41.6	61.4
2	4.0	1.9	1.0	5.4	58.9	38.5	72.3	43.9	60.5	39.9
3	50.9	39.5	44.6	29.9	57.3	46.9	39.6	40.0	30.1	34.9
4	6.0	84.9	6.0	84.9	16.5	65.5	20.0	70.9	17.4	71.8
5	10.9	20.4	31.9	39.4	36.5	34.6	14.5	20.8	7.8	16.4
6	1.1	37.9	1.4	42.8	5.8	38.0	69.3	25.5	17.0	45.3

	**Explicit wanting High Fat**	**Explicit wanting Low fat**	**Explicit wanting High Fat**	**Explicit wanting Low fat**	**Explicit wanting High Fat**	**Explicit wanting Low fat**	**Explicit wanting High Fat**	**Explicit wanting Low fat**	**Explicit wanting High Fat**	**Explicit wanting Low fat**

1	50.0	57.5	56.0	74.0	49.6	66.6	28.1	61.9	40.9	59.8
2	1.9	2.8	1.3	1.8	6.5	13.8	3.0	3.9	58.9	41.3
3	50.8	39.9	41.4	30.0	57.0	46.6	37.6	41.3	27.9	33.4
4	3.1	81.9	3.1	81.9	13.4	59.3	17.0	72.3	18.4	73.8
5	3.8	10.1	17.1	45.5	13.6	19.6	6.6	13.8	10.9	5.4
6	1.0	31.9	1.0	33.0	1.1	33.5	23.8	3.8	1.8	21.0

	**Implicit wanting High Fat**	**Implicit wanting Low fat**	**Implicit wanting High Fat**	**Implicit wanting Low fat**	**Implicit wanting High Fat**	**Implicit wanting Low fat**	**Implicit wanting High Fat**	**Implicit wanting Low fat**	**Implicit wanting High Fat**	**Implicit wanting Low fat**

1	-9.0	9.0	4.7	–4.7	–3.7	3.7	–37.0	37.0	–12.8	12.8
2	–40.2	40.2	–47.9	47.9	–14.2	14.2	–39.7	39.7	–9.3	9.3
3	3.9	–3.9	–2.0	2.0	7.1	–7.1	–13.0	13.0	–18.2	18.2
4	–34.2	34.2	–34.2	34.2	–42.9	42.9	–37.8	37.8	–37.0	37.0
5	–39.9	39.9	–30.6	30.6	–14.0	14.0	–36.5	36.5	–24.9	24.9
6	–43.8	43.8	–41.0	41.0	–40.5	40.5	24.3	–24.3	–25.2	25.2

*Data for Patient 7 were missing.*

## Discussion

The primary objective of this study was to investigate the feasibility and safety of DBS for severe enduring AN. There were no serious adverse events, and all patients elected to continue DBS after the initial year. Given the intervention is invasive and carries risk all the study participants were carefully selected to have high levels of capacity and general cooperation, and subject to a rigorous ethical assessment prior to inclusion by an independent psychiatrist/ethicist who acted as advocate for them throughout the study (Pugh et al., 2018). This intervention would not, we feel be suitable for patients with emotionally unstable, impulsive traits. Unfortunately patient 4, who had severe depression, became nihilistic to the point of not charging his DBS when depression was very severe.

The secondary objective was to investigate the efficacy of DBS on eating disorder psychopathology. In this pilot study of deep brain stimulation to the NAcc, in severe AN, three patients responded, with statistically significant reduction in EDE and CIA scores, and four did not. Bearing in mind that these patients had severe and enduring anorexia, had failed all other interventions and been severely impaired by disease for the majority of their adult lives, this is scientifically and clinically remarkable. Perhaps most importantly, this intervention was described as life changing by the responder group and their families. Interestingly, two of the responders showed changes in their food preferences, shifting away from a maladaptive preference for low fat foods toward a more adaptive preference for energy dense foods. However, no participants experienced over eating binges or the urge to binge, and in general, individuals felt more, rather than less, in control of their eating choices.

Of course, the underlying drivers of variability between responders and non-responders cannot be fully established from this small pilot study. Despite sharing the same diagnosis of SE-AN, the participants differ in multiple ways, for example age, duration of illness and psychiatric comorbidities. However, our results do give some clues on what may predict response, and raise important questions for further investigation.

### Possible Predictors of Response

#### Premorbid Childhood Obsessive–Compulsive Disorder

This study chose a deep brain target which showed promise in alleviating obsessions and compulsivity in OCD (Denys et al., 2010, 2020; Tyagi et al., 2019), and a 75% response rate is reported in treatment resistant OCD trials (Bergfeld et al., 2021). It is notable that in the three responders, OCD diagnosis in childhood pre-dated the onset of AN, whereas the non-responders did not report a premorbid childhood onset OCD. Plausibly, it may be that the DBS impacted primarily on the OCD/compulsivity neural circuits on which their AN thrived. This may have permitted the dwindling of AN pathology, increasing flexibility and their ability to engage with treatment for AN. There is a notable absence of ED treatment response in patient 3 and she had an OCD diagnosis, but her OCD developed later, after the development of AN in her mid-twenties.

#### Stimulation Intensity

A further factor potentially contributing to a lack of full response is sub-optimal stimulator intensity in patients 3 and 6. The stimulator setting schedule was based on a prior study in an OCD cohort (Denys et al., 2010). In this study, the two aforementioned participants were notably unable to tolerate stimulation above 3.5V (6) and 3.75 V (3) due to side effects. Whilst it is possible that this might have hampered their response, others who were stimulated at higher voltages also failed to respond (see Table 2 for full details of stimulator settings). Indeed, Ramasubbu et al. (2018) concluded that the locus of stimulation is more important than stimulation intensity in determining treatment response of DBS for treatment resistant depression. The authors found no significant difference in mean stimulation parameters between responders and non-responders, suggesting optimal intensity is likely to be highly individualized.

#### Network Connectivity

Active contact selection determines this locus of stimulation and has previously been demonstrated to predict treatment response in Parkinson’s disease and OCD (Horn et al., 2017; Li et al., 2020). Since each participant’s neuroanatomy will differ with respect to white matter connectivity from the NAcc, this individualized variability may also account for treatment response variability (Alhourani et al., 2015; Duriez et al., 2020). This can be investigated using diffusion tensor imaging (DTI) analysis to estimate axonal organization and quantify cortical connectivity and has been used previously for personalization of targets prior to electrode implantation (Tyagi et al., 2019). Furthermore, in discussing the future of personalized neurostimulation, Figee and Mayberg recommend that analysis of fiber connectivity using diffusion tensor imaging, as opposed to precise neural regions, should be implemented for target selection (Figee and Mayberg, 2021).

### Defining Response

The strict definition of response used here is guided by the OCD literature, where a >35% decrease YBOCS score compared to baseline was considered to be a response (Denys et al., 2010, 2020; Lipsman et al., 2017). In this study, the primary outcome measure was the EDE which is a structured interview and has four subscales: restriction, weight concern, shape concern and eating concern. Treatment response was defined as (a) > 35% reduction in EDE from immediately prior to DBS switch on (not preoperative baseline) to EDE at 12 months. These criteria were chosen to be broadly in line with prior DBS studies using the YBOCS in OCD cohorts (Denys et al., 2010). By taking our starting point to measure response as immediate pre switch on, we attempt to control for any effect of the pre op assessment, and or the operation itself, on psychopathology.

#### To What Extent Is Body Mass Index an Index of Recovery?

In clinical studies participants tend generally to be in a naturalistic “treatment as usual” setting, and subject to external factors as part of their recovery journey. These factors all confound using BMI as an index of recovery, which can be artificially conflated by refeeding and mask underlying psychopathology. We chose not to use BMI as the sole primary outcome because it is a poor reflection of shifts in eating disorder psychopathology; for example, the onset of binge eating, or admission into inpatient refeeding programs, may increase BMI, but can leave core eating disorder pathology and the urge to restrict unchanged and sometimes even worsened. For example, patient 3 had a BMI of 18 as a result of being in inpatient weight restoration, yet had extremely high levels of eating disorder and OCD pathology and was unable to eat outside an inpatient setting. That said, we do not discount the importance of BMI on overall outcome and eventual recovery. Full recovery after many years AN, is likely to be gradual and take time. We predict BMI should improve in responders as a longer term effects of DBS reductions in eating disorder psychopathology.

At the end of the 12 months stimulation period, BMI was not significantly increased compared to baseline in the responder group. While weight restoration does not necessarily define recovery from AN, it is possible that nutritional status and intensive support with concurrent weight restoration be advised in future studies of DBS/neuromodulation, to optimize the chance of weight gain.

The first published case series targeted the subcallosal cingulate with a 50% response rate at one year (Lipsman et al., 2017) and no further improvements at 3 years follow-up. The authors primarily focused on BMI as a measure of ‘recovery,’ rather than eating disorder psychopathology. Two recent DBS case series have been published, which have targeted the NAcc in an AN patient population (Liu et al., 2020; Villalba Martínez et al., 2020) and both focused on BMI as a primary outcome measure. Neither study reports on eating disorder psychopathology and neither include a blind on vs. off period, making it impossible to establish changes in eating disorder psychopathology, or if any effects are a genuine attributable to DBS. Our study, reported here attempts to overcome these important limitations by inclusion of a rigorous definition of response, a blind DBS on off period, and detailed measurement of eating disorder psychopathology alongside a rigorous ethical gold standard.

In China, Liu et al. (2020) recently published a two-year case series of 28 young women with AN over 3 years duration. Participants were a mix of restrictive AN (13) and binge-purge subtype (15). The authors reported increased BMI following stimulation, an improvement in OCD, anxiety and depression scores. However, they did not directly assess core eating disorder symptoms and the authors acknowledge that those with a short duration of illness may have recovered without intervention. Ethical questions have been raised over whether participants with such a short illness history should be included in a DBS trial (Cleary et al., 2015; Stevens and Gilbert, 2021).

Villalba Martínez et al. (2020) recently published their 6-month data from a longitudinal case-series of eight participants with AN (seven were restrictive subtype). They tailored the location of stimulation on the basis of comorbid phenotype based on the success of previous DBS studies in treating the comorbid depression/OCD. Three participants with depression received DBS to the subcallosal cingulate. Whilst four participants with an anxiety disorder, received DBS to the NAcc (Villalba Martínez et al., 2020). At 6 months, the authors reported a modest but non-significant increase in BMI and subjective improvement in quality of life but the effect on ED pathology are not documented.

### Refining Response: The Importance of the Blind On/Off Month

During the double-blind month off period, the two full responders (patients 1 and 2), experienced a temporary exacerbation of eating disorder symptomatology, depression and anxiety which reversed when the stimulator was switched back on, such that improvement continued until the end of the protocol. This suggests a genuine stimulation effect in these participants. The on/off stimulation month coincided with a personally difficult month for patient 6, which might have masked any genuine changes. To our knowledge this is the first double-blind DBS protocol in AN, although there have been several prior double-blind studies in OCD (Denys et al., 2010; de Haan et al., 2017; Tyagi et al., 2019). In an open-label study, obsessive/compulsive symptoms recurred when stimulation was switched off (McLaughlin et al., 2013).

### Strengths and Limitations

Although the study is limited by small sample size it is the first study in AN to include a blind on vs. off DBS phase and to measure eating disorder psychopathology in detail over time; the importance of the detailed data and rigorous outcome measures cannot be underestimated, and these patients are now in ongoing follow-up as time course of change with DBS cannot yet be determined. Detailed MEG scan findings and neuropsychological findings are reported in separate papers^1,2^.

### Future Directions

The inconsistent response rate in DBS studies in OCD and Depression cohorts always raises discussion about patient selection criteria and optimum deep brain targets and parameters for stimulation (Roet et al., 2020; Figee and Mayberg, 2021). Research in AN is hampered by a scarcity of DBS studies and the complexity of clinical presentation, including numerous comorbidities. Recent evidence suggests proactive inhibition (Bartholdy et al., 2017) which is impaired in OCD in line with severity, is altered by NAcc DBS in OCD patients (Sildatke et al., 2021). Given responsiveness in those comorbid early onset OCD, proactive motor inhibition may be an underlying mechanism worthy of investigation in future AN studies As more research is undertaken and the neural circuitry underpinning AN is further characterized as it has been in OCD and depression, a more personalized and symptom-based approach will be possible. Furthermore, with these advancements, we may be able to stimulate or perturb neural networks rather than individual areas. Closed-loop DBS systems simultaneously record and stimulate neural activity, allowing the stimulation to be adjusted according to disease-specific neural biomarkers with possible integration of multiple feedback sites and artificial intelligence to fine-tune programming (Krauss et al., 2021).

An important recent development in DBS technology are electrodes with multiple independently controllable contacts (Weerasinghe et al., 2021). This has been used successfully in a case study using closed-loop neuromodulation to treat an individual with chronic depression. They implanted a chronic deep brain sensing and stimulation device and implemented a biomarker-driven closed-loop therapy which was triggered when symptoms were elevated (Scangos et al., 2021). In future, a top–down, non-invasive approach, which targets cortical regions may be taken instead. In OCD, abnormal beta–gamma neurophysiology of the orbitofrontal–striatal circuitry is observed during reward processing. Top–down stimulation using transcranial direct current stimulation targeting the orbitofrontal cortex improves OCD symptomatology (Grover et al., 2021).

## Conclusion

This is first study of DBS of for severe enduring AN to include a blinded off period and detailed investigation of the effects of DBS on eating disorder psychopathology *per se*. Although the study is limited by small sample size its strength is in detailed longitudinal data on all aspects of eating psychopathology and comorbid symptoms. We found that patients with premorbid early onset OCD prior to AN onset appear to benefit the most from DBS. For them it was experienced as a game-changing intervention. In contrast, all non-responders had a later onset of AN over the age of 18, and only one had comorbid OCD which was also of later onset. Differences in underlying neural circuitry and or electrode placement may also contribute to lack of response to DBS. Concurrent engagement in intensive weight restoration program is advisable to accompany and facilitate DBS induced neurogenesis and change. All patients are in active follow-up and all elected to continue DBS over the next 3 years to establish future course. It remains possible non-responders may yet show improvement during the longer term.

## Data Availability Statement

The original contributions presented in the study are included in the article/Supplementary Material, further inquiries can be directed to the corresponding author/s.

## Ethics Statement

The studies involving human participants were reviewed and approved by NRES: South Central—Oxford A Research Ethics Committee (REC) Ref: 13/SC/0267. Informed written consent was in accordance with the Declaration of Helsinki. The patients/participants provided their written informed consent to participate in this study.

## Author Contributions

RP led on psychiatric aspects of the study, designed the protocol, information sheets, and wrote REC and grant applications. TA led on all surgical aspects of the study. AG contributed invaluable surgical support. RP, BP, JE, and JS acquired the data. JS, RP, JE, and AG drafted the manuscript. All authors read and approved the final manuscript.

## Author Disclaimer

The views expressed are those of the authors and not necessarily those of the NHS, the NIHR, or the Department of Health’.

## Conflict of Interest

The authors declare that the research was conducted in the absence of any commercial or financial relationships that could be construed as a potential conflict of interest.

## Publisher’s Note

All claims expressed in this article are solely those of the authors and do not necessarily represent those of their affiliated organizations, or those of the publisher, the editors and the reviewers. Any product that may be evaluated in this article, or claim that may be made by its manufacturer, is not guaranteed or endorsed by the publisher.

## Footnotes

1 Prangnell, S. J., Scaife, J. C., Aziz, T. Z., and Park, R. J. (under review). Neuropsychological outcome from nucleus accumbens deep brain stimulation for severe enduring anorexia nervosa.

2 Braeutigam, S., Scaife, J. C., Aziz, T. Z., and Park, R. J. (under review). A longitudinal magnetoencephalographic study of the effects of deep brain stimulation on neuronal dynamics in severe anorexia nervosa.

## Abbreviations

AN: Anorexia Nervosa
BMI: Body Mass Index
CI: Chief Investigator
DBS: Deep-brain stimulation
DTI: Diffusion Tensor Imaging
NAcc: Nucleus Accumbens
OCD: Obsessive–Compulsive Disorder.
