# Supplementary material for: Deep Brain Stimulation of the Nucleus Accumbens in Severe Enduring Anorexia Nervosa: A Pilot Study
Source: Front Behav Neurosci. 2022 Apr 27;16:842184. doi: 10.3389/fnbeh.2022.842184 (PMC9094709; doi:10.3389/fnbeh.2022.842184)
Supplement: Supplementary file 1 [file Table_1.docx]

**Supplementary Table 1**

Side effects reported on the online DBS-Side effects questionnaire during protocol period. Short term side effects resulted from DBS operation / switch on, long term side effects persisted during the protocol period.

| Patient number | Short-term side effects | Long term side effects |
| --- | --- | --- |
| 1 | scalp pain, headache, neck pain, trouble concentrating, nausea, appetite loss | scalp pain |
| 2 | headache, skin redness, acute mood change, trouble concentrating, toothache | none |
| 3 | headache | none |
| 4 | scalp pain, headache, neck pain, neck stiffness, itching, burning sensation, skin Redness, trouble concentrating, acute mood change, altered sensation on scalp, difficulty with urination, sleepiness | Scalp pain, headache trouble concentrating, and sleepiness |
| 5 | scalp pain, headache, tingling, trouble concentrating, sleepiness, mood change | none |
| 6 | headache, neck pain, itching, skin redness, sleepiness, trouble concentrating, severe shoulder pain, subjective short term memory impairment and word finding difficulties, change in taste of food and drink, nausea, vertigo, travel sickness | headache, neck pain, sleepiness, trouble concentrating, subjective short term memory impairment, nausea, vertigo, travel sickness |
| 7 | sleepiness, headache | none |
